# Cellular injury and neuroinflammation in children with chronic intractable epilepsy

**DOI:** 10.1186/1742-2094-6-38

**Published:** 2009-12-19

**Authors:** Jieun Choi, Douglas R Nordli, Tord D Alden, Arthur DiPatri, Linda Laux, Kent Kelley, Joshua Rosenow, Stephan U Schuele, Veena Rajaram, Sookyong Koh

**Affiliations:** 1 Department of Pediatrics, Northwestern University Children's Memorial Hospital, Chicago, IL, USA; 2 Department of Pediatrics, Seoul National University Boramae Hospital, Seoul, Korea; 3 Department of Neurosurgery, Northwestern University Children's Memorial Hospital, Chicago, IL, USA; 4 Department of Neurosurgery, Northwestern Memorial Hospital, Northwestern University, Chicago, IL, USA; 5 Department of Neurology, Northwestern Memorial Hospital, Northwestern University, Chicago, IL, USA; 6 Department of Pathology, Northwestern University Children's Memorial Hospital, Chicago, IL, USA

## Abstract

**Objective:**

To elucidate the presence and potential involvement of brain inflammation and cell death in neurological morbidity and intractable seizures in childhood epilepsy, we quantified cell death, astrocyte proliferation, microglial activation and cytokine release in brain tissue from patients who underwent epilepsy surgery.

**Methods:**

Cortical tissue was collected from thirteen patients with intractable epilepsy due to focal cortical dysplasia (6), encephalomalacia (5), Rasmussen's encephalitis (1) or mesial temporal lobe epilepsy (1). Sections were processed for immunohistochemistry using markers for neuron, astrocyte, microglia or cellular injury. Cytokine assay was performed on frozen cortices. Controls were autopsy brains from eight patients without history of neurological diseases.

**Results:**

Marked activation of microglia and astrocytes and diffuse cell death were observed in epileptogenic tissue. Numerous fibrillary astrocytes and their processes covered the entire cortex and converged on to blood vessels, neurons and microglia. An overwhelming number of neurons and astrocytes showed DNA fragmentation and its magnitude significantly correlated with seizure frequency. Majority of our patients with abundant cell death in the cortex have mental retardation. IL-1beta, IL-8, IL-12p70 and MIP-1beta were significantly increased in the epileptogenic cortex; IL-6 and MCP-1 were significantly higher in patients with family history of epilepsy.

**Conclusions:**

Our results suggest that active neuroinflammation and marked cellular injury occur in pediatric epilepsy and may play a common pathogenic role or consequences in childhood epilepsy of diverse etiologies. Our findings support the concept that immunomodulation targeting activated microglia and astrocytes may be a novel therapeutic strategy to reduce neurological morbidity and prevent intractable epilepsy.

## Background

The deleterious contribution of inflammation has been well established for a growing number of neurological disorders such as cerebral ischemia, traumatic brain injury, multiple sclerosis, and HIV encephalitis [1]. Involvement of inflammation in the pathogenesis of epilepsy and seizure-induced brain damage, however, has only recently been explored [2,3]. Active inflammation has been detected not only in prototypical inflammatory epilepsies such as Rasmussen's encephalitis or limbic encephalitis, but also in patients with pharmacoresistant epilepsy of diverse causes [2-7]. Pro-inflammatory cytokines, NF-κB, interleukin (IL)-1β and its signaling receptor IL-1R1 are highly expressed by neurons and glia in temporal lobe epilepsy [3,4], focal cortical dysplasia [5], glioneuronal tumor [6], and in tuberous sclerosis complex [7]. Increased levels of cytokines such as IL-6 [8], IL-1β [9] and IL-1-receptor antagonist [10] have also been detected in plasma and cerebrospinal fluid from patients with recent seizures without evidence of infection. Whether cytokines contribute directly to pathogenesis of seizures and chronic epilepsy, or the high levels of cytokines merely reflect activation following seizures, however, cannot be determined from these clinical observations. The causative role of neuroinflammation in the development of chronic epilepsy remains unclear.

In many animal models of epilepsy, acute seizures cause glial activation and increased cytokine production [11-13]. Experimentally induced seizures trigger a rapid inflammatory response in brain areas recruited in the onset and propagation of epileptic activity [14]. Members of the Toll-like receptor (TLR) family are significantly up-regulated by seizures in microglia, and their engagement leads to transcriptional activation of cytokines, chemokines, MHC class I and II and costimulatory molecules [11]. The activated glia and elevated cytokines, in turn, have been demonstrated to contribute to seizure-related hippocampal pathology, such as neuronal death, neuronal birth, reactive gliosis and mossy fiber sprouting [15]. IL-1β increases glutamatergic neurotransmission and lowers the peak magnitude of GABA-mediated currents [16], contributes to generation of fever-induced seizures [17] and prolongs the electroencephalographic seizure duration [18]. Available experimental data thus suggest that seizure-induced glial activation and up-regulation of pro-inflammatory cytokines can lead to neuronal excitability and neuronal injury either directly by interacting with glutamatergic neurotransmission, or indirectly by activating gene transcription.

Microarray analyses in our laboratory have shown that genes coding for classical inflammatory mediators - complement pathway, cytokines and glial markers - are acutely increased in both immature and mature rat hippocampus after kainic acid (KA)-induced seizures [19]. Inflammation-related genes are the functional group that are most significantly up-regulated acutely, and they remain elevated 10 days after seizures in mature, but not in immature, hippocampus. Persistent inflammation occurring only in older animals (that show seizure-induced cell death and subsequently develop spontaneous recurrent seizures) implies a causative role of chronic active inflammation in epileptogenesis and seizure-induced cell death. These age-dependent increases are reflected in tissue microglial activation [20,21]. In order to determine whether active inflammation and neuronal degeneration occur in pediatric epilepsy, we processed brain tissue from patients with intractable childhood epilepsy. We quantified DNA fragmentation for cell injury and looked for the evidence of inflammation by astrocyte and microglia staining and by cytokine assay.

## Methods

### Patient information

Thirteen patients with chronic intractable epilepsy who underwent epilepsy surgery from January 2006 to June 2007 were included in order of operation (Table 1). Normal-appearing control cortex was obtained at autopsy from eight patients without seizures or other neurological diseases. All autopsies were performed within 12 hours after death. Extensive pre-surgical evaluation included video-EEG-monitoring, MRI, and electrocorticography, as well as ictal and interictal SPECT and FDG-PET-imaging as needed. None of our patients had a grid placement or other invasive monitoring prior to functional hemispherectomy or lobectomy. This study was approved by Institutional Review Board at the Children's Memorial Research Center and at the Northwestern Memorial Hospital.

**Table 1:** Clinical findings in epilepsy patients.

		Age (yr) at		Seizure							Post-op F/u					
										
No	Sex	Op	Onset	Dur (yr)	Freq/month	FHx	Feb sz	MR	ADHD	HP	Dur (yr)	Sz outcome	Epileptogenic focus		Diagnosis	
1	F	2.9	2.5	0.4	30-450	--	--	†	--	R	3.0	sz-free	L hemisphere		cortical dysplasia	

2	M	2.4	1.5	0.9	30-120	†	--	†	--	--	2.0	sz-free	L.inf. temporal		cortical dysplasia	

3	M	4.4	0.2	4.2	60	†	--	†	--	L	1.8	75% ↓	R frontal, central mid-temporal		cortical dysplasia	

4	M	3	0.1	2.9	1-4	--	--	†	--	L	1.6	sz-free	R post temporo-occipital		cortical dysplasia	

5	M	7.5	4.8	2.7	150-300	--	--	†	--	R	2.7	sz-free	L hemisphere		encephalomalasia stroke	

6	F	19.5	3	16.5	4-8	†	--	†	--	L	2.5	sz-free	R parieto-temporo-occipital		encephalomalasia stroke	

7	F	9.7	4	5.7	90-120	N/A	--	†	†	L	2.3	sz-free	R hemisphere		encephalomalasia traumatic injury	

8	F	13.2	8	5.2	120	†	--	--	--	--	2.1	sz-free	R frontal		encephalomalasia tumor resection	

9	M	28	13	15	30-60	--	--	†	--	--	2.6	sz-free	R temporal		encephalomalasia traumatic injury	

10	F	9	7	2	1-4	--	†	--	†	--	1.6	sz-free	L ant-middle temporal		hippocampal sclerosis	

11	F	9.2	5.8	3.4	500	--	†	†	†	L	2.1	sz-free	R hemisphere		Rasmussen's encephalitis	

12	M	17	9	8	30-60	--	--	--	†	--	2.9	sz-free	L frontal		cortical dysplasia	

13	F	18	12	6	4-16	†	--	†	--	L	1.6	sz-free	R frontal		cortical dysplasia	

Op = operation; yr = year; Dur = duration; freq = frequency; FHx = (+) family history of epilepsy; Feb sz = febrile seizure; MR = mental retardation; ADHD = attention deficit hyperactivity disorder; HP = hemiparesis; F/U = follow-up; F = female; M = male; Sz = seizure; L = left; R = right; inf = inferior; post = posterior; N/A = not applicable.

### Brain tissue processing

Fixed or frozen brain tissue was obtained at the time of resection in the operating room. The cortical gray matter (cortex) was rapidly dissected and cut into approximately 5 mm^3 ^pieces and immediately frozen in cooled isopentane over dry ice, and stored at -80°C. A block of resected tissue including both cortex and subcortical white matter was emersion fixed in 4% paraformaldehyde solution in 100 mM PBS, pH 7.4. After fixation, tissue was cryoprotected in 30% sucrose and 50 μm-thick serial sections were cut and collected. A total of 22 regions from 11 patients (Table 2) were processed for immunohistochemistry as described below. Adjacent sections were processed for *in situ *end labeling (ISEL) nick translation to detect DNA fragmentation as a marker for cell injury and Fluoro-Jade B staining to detect degenerating cells.

**Table 2:** Brain tissue information

				Tissue		Data used for analysis		
				
**Patient No**.	Age	Diagnosis	Brain Region	Fixed	Frozen	IHC	Cytokine	Correlation
1	2.9	cortical dysplasia	frontal	†		†		†
			temporal	†	†		†	†

2	2.4	cortical dysplasia	anterior temporal	†	†	†		†
			inferior temporal	†	†		†	†

3	4.4	cortical dysplasia	temporal	†	†	†	†	†

4	3	cortical dysplasia	temporal	†	†			†
			parietal	†				†
			occipital	†	†	†	†	†

5	7.5	encephalomalacia	temporal	†	†	†	†	†
			occipital	†				†

6	19.5	encephalomalacia	anterior temporal	†				†
			lateral temporal	†	†	†	†	†
			temporal	†	†			†
			occipital	†				†

7	9.7	encephalomalacia	frontal	†			†	†
			temporal	†	†	†		†

8	9.2	encephalomalacia	frontal	†	†	†	†	†

9	28	encephalomalacia	lateral temporal	†	†	†	†	†

10	9	hippocampal sclerosis	temporal	†	†	†	†	†

11	9.2	Rasmussen's encephalitis	frontal		†		†	†
			superior temporal	†	†	†		†
			perisylvian/insular	†				†

12	17	cortical dysplasia	temporal		†		†	†

13	18	cortical dysplasia	temporal		†		†	†

Control No.								

1	15	asthma	temporal	†		†		†

2	0.9	pneumonia	frontal	†		†		†
			temporal	†		†		†
			parietal	†		†		†

3	0.5	sepsis	frontal	†		†		†
			temporal	†		†		†

4	64	hypertension, diabetes mellitus	frontal	†	†	†	†	†
			occipital	†	†		†	†

5	79	sepsis	frontal	†	†	†	†	†
			occipital	†	†		†	†

6	15.1	Vehicle accident (chest)	Frontal, temporal	†	†	†		†

7	8	Cardiac arrhythmia	Frontal, temporal	†	†	†		†

8	31.8	Alcohol intoxification	Frontal, temporal	†	†	†		†

Op = operation; yr = year; Dur = duration; freq = frequency; FHx = (+) family history of epilepsy; Feb sz = febrile seizure; MR = mental retardation; ADHD = attention deficit hyperactivity disorder; HP = hemiparesis; F/U = follow-up; F = female; M = male; Sz = seizure; L = left; R = right; inf = inferior; post = posterior; N/A = not applicable.

### Immunohistochemistry and in situ end labeling nick translation histochemistry (ISEL)

Antibodies to glial fibrillary acidic protein (GFAP, DAKO, Glostrup, Denmark) for astrocytes, neuronal nuclear protein (NeuN, Chemicon, Temecula, CA) for neurons, and CD68 (KP1, DAKO) for microglia and macrophages were used. For immunohistochemistry, free floating sections were processed using the avidin-biotin peroxidase method with 3, 3-diaminobenzidine. To examine the spatial relationship between neurons, astrocytes and microglia, double or triple immunofluorescence was performed. Briefly, the sections were mounted on premium microscope slides (Fisher Scientific, Pittsburgh, PA) and were incubated with anti-GFAP or anti-CD68 at 4°C overnight followed by biotinylated secondary antibodies. The sections were then treated with HRP-conjugated streptavidin followed by tyramide-Alexor Flour (Molecular Probe, Eugene, OR) for 1 hour at room temperature. After washing, the sections were treated with anti-NeuN followed by fluorescein anti-mouse antibody. To quench endogeneous autofluorescence, tissue sections were treated with 1% Sudan black in 70% ethanol for 5 min and differentiated with 70% ethanol. Specimens were examined in a Zeiss LSM 5.0 confocal microscope (Zeiss, Thornwood, NY). For detection and quantification of DNA fragmentation, sections were processed for ISEL as previously described [22]. To determine the cell types showing DNA fragmentation, we performed triple immunofluorescent staining for neuron, microglia or astrocytes using TSA-amplification with ISEL. Adjacent sections were processed for Fluoro-Jade B histochemistry as previously described [23] to confirm the detection of degenerating neurons by ISEL staining.

### Quantitative analysis of immunoreactive profiles

At least 5 representative fields from 5-7 tissue sections obtained from different cortical regions of each patient were evaluated (Table 2). The cortex and white matter were analyzed and quantified separately. Images of at least 500 cells were captured digitally at 20x magnification, converted to gray scale, and areas of specific immunoreactive cells were highlighted at a threshold held constant for all specimens and quantified using an image analysis system, MetaMorph (v. 6.1, Universal Imaging Corp., Downingtown, PA) as previously described [24]. An average percent area above the threshold was calculated per brain region and comparison was made between individual patients or between epilepsy sub-groups of common etiology and controls. For quantification of cellular subtypes showing DNA fragmentation, three sections from three patients proven to have high DNA fragmentation were selected and five images each from cortical area were captured digitally at 25x magnification in a confocal microscope. Percent of cells double-labeled with NeuN, GFAP or CD68 and fragmented DNA over total number of ISEL-positive cells was calculated.

### Measurement of cytokines and chemokines

Frozen cortices from all patients (*n *= 13) and available controls (*n *= 2) (Table 2) were homogenized with PM buffer from a total protein extraction kit (Chemicon, Temecula, CA), then centrifuged at 4°C and stored at - 80°C. Total protein concentration was calculated using the BCA assay kit (Pierce, Rockford, IL). Levels of proinflammatory cytokines including interferon (IFN)-γ, IL-1β, IL-2, IL-6, IL-8, IL-12p70, TNF-α, granulocyte-macrophage colony stimulating factor (GM-CSF), anti-inflammatory cytokine IL-10, and chemokines including eotaxin, eotaxin-3, IFN-inducible protein 10 (IP-10), MCP-1, MCP-4, macrophage derived chemokine (MDC), macrophage inflammatory protein 1β (MIP-1β), and thymus and activation-regulated chemokine (TARC) were measured using ELISA-based commercially available kits (Meso-Scale Discovery, MSD, Gaithersburg, MD). Samples were analyzed in duplicates and compared with controls. Plates were analyzed using the SECTOR Imager 2400 (MSD).

### Statistical Analysis

A one-way analysis of variance (ANOVA) was used to compare immunoreactive profiles for astrocytes, microglia and DNA fragmentation between controls and patients. For multiple comparison groups, Tukey's correction was applied (GraphPad Prism v. 4.0, GraphPad Software Inc., San Diego, CA). Nonparametric Spearman's rank correlation coefficient was calculated to find significant correlations between glia activation, DNA fragmentation, different clinical variables and cytokine levels. Student t-test was used to compare age of onset and duration of seizures between cortical dysplasia and encephalomalasia. Nonparametric Mann-Whitney test was used to compare cytokine and chemokine levels between controls and patients. We included all available quantification data for correlation, while select representation from each patient was used for group comparison between controls and patients (see Data used for analysis, Table 2). Values are expressed as mean ± SEM and significance was defined as *p *< 0.05 for all tests.

## Results

### Patient characteristics and pathology

**Figure 1:**
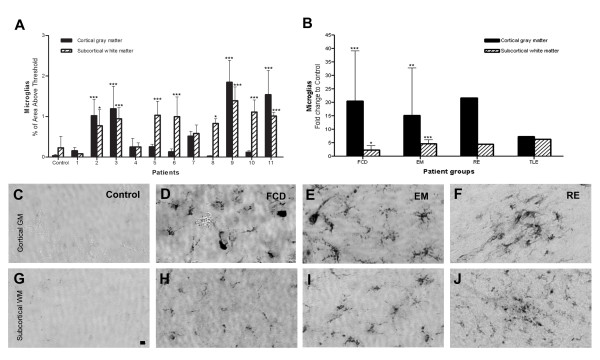
**Quantification of CD68 immunoreactive microglia in the cortex (cortical GM) and white matter (subcortical WM). **(**A**) CD68-immunoreactivity in individual patients (patients No. 1 to 11) and controls (*n *= 5). Significant microglial activation was noted in 8/11 patients (73%, *p *< 0.05). (**B**) Fold changes in the CD68-immunoreactivity in epilepsy subgroups compared to controls. Note the magnitude of microglia activation (nearly 20-fold increase in the cortex) in FCD and EM is comparable to RE. (**C-J**) CD68-immunoreactiviy in the cortex (upper panel) and white matter (lower panel). Controls show very rare microglia (**C, G**). Focal cortical dysplasia (**D, H**) and encephalomalacia (**E, I**) show uniform increase in microglia while Rasmussen's encephalitis (**F, J**) show microglial aggregates. Bar = 20 μm. *p < 0.05, ** *p *< 0.01, *** *p *< 0.001, one-way ANOVA.

Table 1 summarizes the patient's clinical data. The two main causes of chronic intractable childhood onset epilepsy were focal cortical dysplasia (FCD, 6 cases) and encephalomalacia (EM, 5 cases) due to intrauterine middle cerebral artery (MCA) stroke (2 cases), traumatic brain injury (2 cases) and previous resection of giant cell astrocytoma (1 case). There was one case each of Rasmussen's encephalitis (RE) and mesial temporal lobe epilepsy with hippocampal sclerosis (TLE). While both patients with RE and TLE had history of prolonged febrile seizures in early childhood, none of our patients with cortical dysplasia or encephalomalacia had experienced febrile seizures. There was a tendency toward younger age of seizure onset (4.2 years ± 2.1 vs. 6.6 years ± 1.8, *p *< 0.43) and shorter duration of seizures (3.7 years ± 1.2 vs. 9.0 years ± 2.8, *p *< 0.098) for patients with cortical dysplasia compared to encephalomalasia group. Interestingly, microglia activation was detected equally in gray and subcortical white matter in patients with cortical dysplasia while microglia were significantly increased only in subcortical white matter in 3/4 patients with encephalomalasia (Fig. 1). Within the cortical dysplasia group, patients with family history of epilepsy (no. 2 & 3) were noted to show significant activation of microglia while patient 1 and 4 with no family history of epilepsy failed to show significant microglia activation. Patient 1 and 2 with cortical dysplasia were noted to have high amount of fragment DNA in cortical gray matter that is comparable to the patient with Rasmussen's encephalitis. They experienced relatively short duration of epilepsy (0.7 years), but had intense seizures with an average of 280 seizures per month. Encephalomalacia resulted from various etiologies: prenatal middle cerebral artery infarction (5 & 6), nonaccidental trauma (7), tumor removal (8) and a fall with massive intracranial hemorrhage (9). There was significant DNA fragmentation in all but one patient (9). The only feature that distinguished this patient from other EM patients was relatively late onset of epilepsy (13 years vs. 5). Our 9 year old TLE patient with hippocampal sclerosis had no risk factors other than febrile status epilepticus and showed no DNA fragmentation as previously noted in adult TLE patients [25]. All but three of our patients had mental retardation. At the most recent postoperative follow-up [1.6-to-3 year (mean 2.2 year)], twelve patients (92%) were seizure-free and one patient had 75% reduction in seizure frequency.

### Diffuse microglial activation in chronic intractable childhood epilepsy

Significant microglial activation was observed in the resected cortices of our patients (*p *< 0.0001) (Fig. 1). Microglial activation involving both cortex and white matter was found not only in a patient with Rasmussen's encephalitis as expected, but also in two patients with focal cortical dysplasia and one with encephalomalacia (patient 7) (Fig. 1A). Notably, microglia were diffusely and uniformly distributed in these patients (Fig. 1D, 1E, 1H and 1I), while they formed localized nodules in the case with Rasmussen's encephalitis (Fig. 1F and 1J). Significant microglial activation was limited to the white matter in 4 patients (4/11, 36%, *p *< 0.001) (Fig. 1A). In control brains, only rare CD68 immunoreactive microglia were detected and scattered around the perivascular spaces (Fig. 1C and 1G).

### Panlaminar reactive astrocytosis in the brains of pediatric epilepsy surgery patients

**Figure 2:**
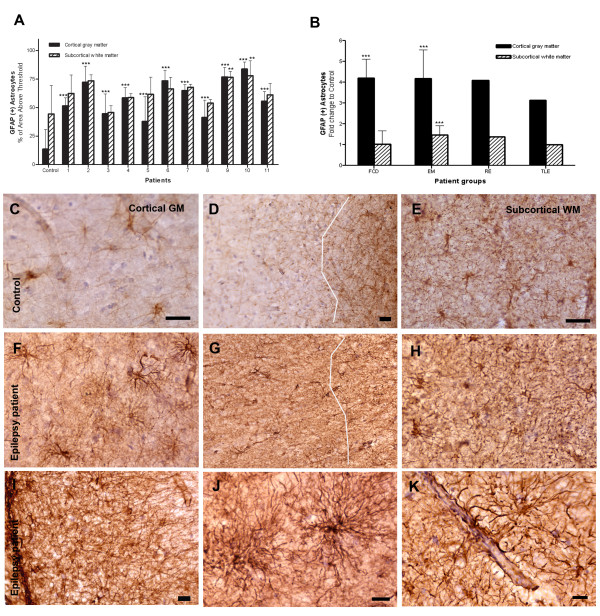
**Quantification of GFAP immunoreactive astrocytes in the cortex and white matter. **(**A**) GFAP-immunoreactivity in individual patients (patients No. 1 to 11) and controls (*n *= 5). (**B**) Fold changes in the GFAP-immunoreactivity in epilepsy subgroups compared to controls. (**C**, **D & E**) Control: cortex, gray-white junction and white matter. (**F, G & H**) Epilepsy case: cortex, gray-white junction and white matter. Notice loss of clear gray-white demarcation in epilepsy case (**G**) compared to control (**D**). (**I-K**) High magnification of an epilepsy case. (**I**) Expanded subpial gliosis. (**J**) Cortical astrocytes with numerous thin and elongated processes, morphologically resembling fibrillary astrocytes. (**K**) Astrocytic end feet adhering to the wall of blood vessel. Bars: C-H = 100 μm; I-K = 20 μm. * *p *< 0.05, ** *p *< 0.01, *** *p *< 0.001 by one-way ANOVA.

All of our patients had diffuse astocyte proliferation that obscured the borders of the gray-white matter junction (*p *< 0.0001) (Fig. 2). Entire cortical layers and white matter were covered by diffuse astrocytic infiltration and were darkened by profuse astrocytic processes, thus rendering the whole brain section dark brown. A thin layer of subpial gliosis was expanded 2- to 5-fold in our patients, thus further obliterating the normal cortical architecture (Fig. 2).

### Close association among neurons, microglia and astrocytes

**Figure 3:**
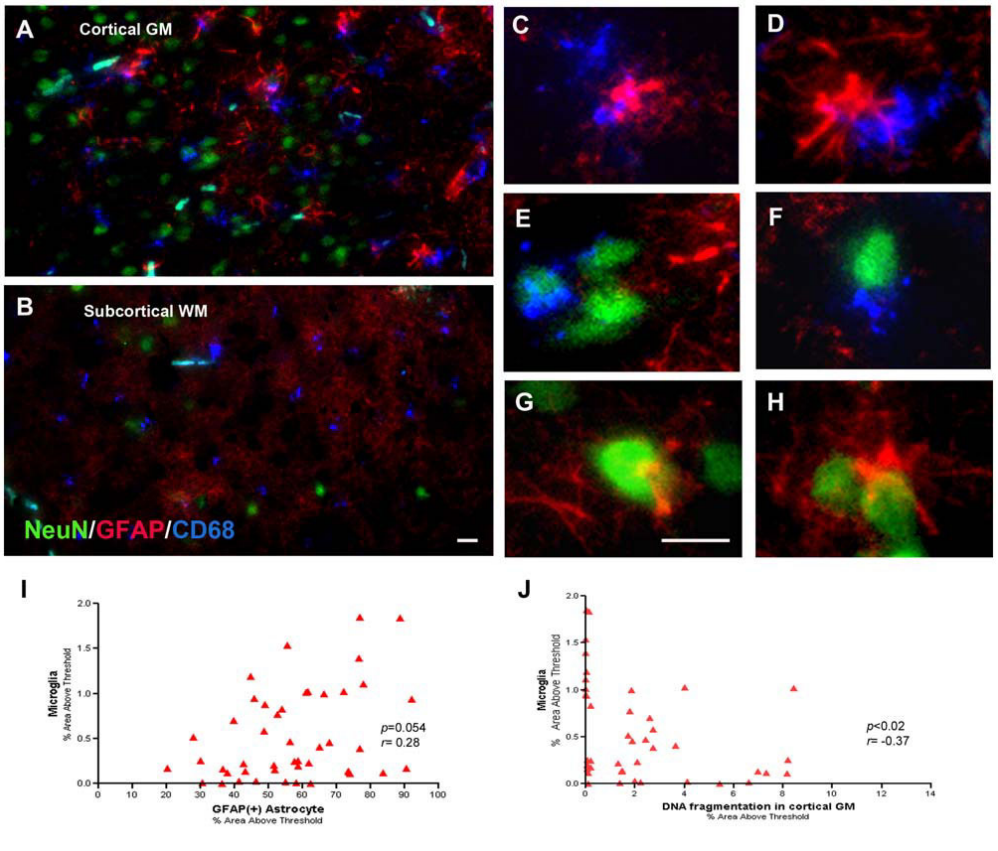
**Triple immunofluorescence confocal images of neurons (green), microglia (blue) and astrocytes (red). **Low magnification view of cortex (**A**) and white matter (**B**). (**C**--**H**) Higher magnification view showing close contact between microglia and astrocytes (**C**, **D**), neurons and microglia (**E**, **F**) and neurons and astrocytes (**G**, **H**). Activated microglia tend to co-localize with astrocytes (*p *= 0.054) (**I**). Activated microglia is inversely correlated with DNA fragmentation (*p *< 0.02, *r *= -0.38) (**J**). Bar = 20 μ m.

Neurons, microglia and astrocytes were often found next to each other and displayed overlapping cell bodies and processes suggesting intimate interaction between these three cell types (Fig. 3). Microglia and astrocytes were often increased in the same region in the cortex and showed trends toward correlated localization (*p *= 0.054) (Fig. 3I). Numerous reactive astrocytes were found in the cortex juxtaposed to neurons, microglia, and blood vessels (Fig. 3B, 3C, 3G and 3H; Fig. 2K). DNA fragmentation was inversely correlated with microglial activation (*r *= -0.37, *p *< 0.02) (Fig. 3J).

### Diffuse DNA fragmentation and degenerating neurons in chronic intractable childhood epilepsy

**Figure 4:**
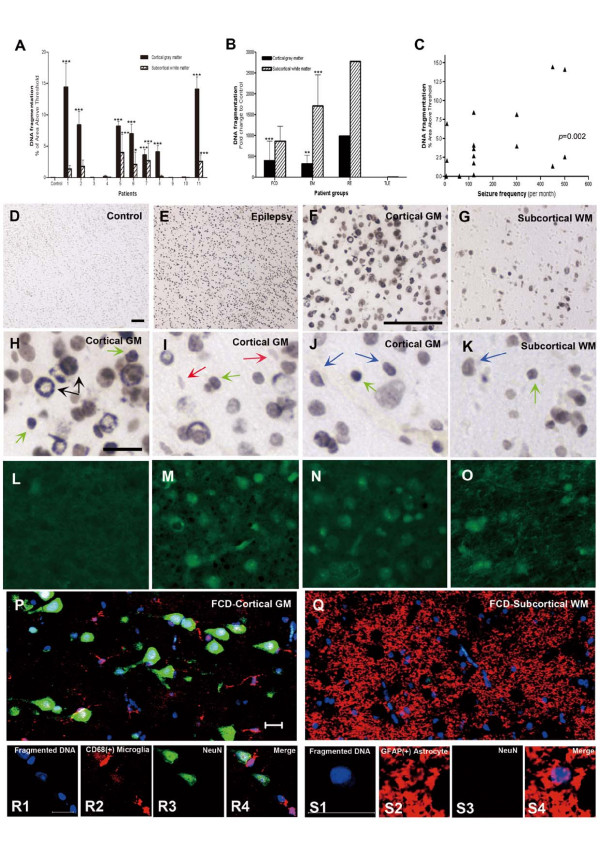
**Quantification and Triple immunofluorescence confocal images of DNA fragmentation in the cortex and white matter**. (**A**) DNA fragmentation in individual patients (patients No. 1 to 11) and controls (*n *= 5). Notice that the majority of our patients [6/10, except RE (No. 11)] showed diffuse cell injury that is comparable to the patient with Rasmussen's encephalitis (No. 11). (**B**) Fold changes in DNA fragmentation in epilepsy subgroups over controls. (**C**) The magnitude of DNA fragmentation is significantly correlated with seizure frequency prior to surgery (*p *= 0.002). (**D**) Low magnification view of a control shows no evidence of DNA fragmentation. (**E**-**G**) Epilepsy case. Low and high magnification of cortex (**F**) and white matter (**G**) show increased DNA fragmentations. (**H**-**K**) Higher magnification view of DNA fragmentation in neurons (black arrows), oligodendrocytes (green arrows) and astrocytes (blue arrows) in cortex (**H**, **I **and **J**) and white matter (**K**). Microglia were spared (red arrows). The identification of different cell types was made based on nuclear morphology. Neurons have large vesicular nuclei and oligodendrocytes have round uniform nuclei while astrocytes have bipolar elongated nuclei with irregular borders. Small rod-shaped nuclei of microglia show only faint staining. (**L-O**) Fluoro-Jade B staining in control (L) and epilepsy cases (M-O). Positive staining in the cortex (M, N) and white matter (O) of epilepsy cases and not in controls are consistent with cell injury noted by *in situ *end labeling of DNA fragmentation. (**P**-**S**) Triple immunofluorescence confocal images of cellular subtypes showing DNA fragmentation in the cortex (**P**, R) and white matter (**Q**, **S**) of focal cortical dysplasia patient; Low magnification (**P**) shows that neurons comprise the majority of cells with DNA fragmentation. Double labeling of neuron and DNA fragmentation is magnified and microglia closely associated with these neurons are also labeled with fragmented DNA (Fig. **R**4). DNA fragmentation (blue); microglia (red); neuron (green). (**P, R**) White matter. DNA fragmentation (blue); astrocyte (red); neuron (green). Low (**Q**) and high magnification (**S**) show double labeling of astrocytes with DNA fragmentation. Bars: D-G = 100 μ m; H-S = 20 μ m. * *p *< 0.05, ** *p *< 0.01, *** *p *< 0.001, one-way ANOVA.

Acute cell injury demonstrated by dense staining of DNA fragmentation and Fluro-Jade B-positive degenerating neurons was significantly increased in epileptic lesions (*p *< 0.0001) (Fig. 4). DNA fragmentation encompassed all cortical layers, as well as the white matter (Fig. 4E, 4F, 4G, 4P and 4Q) and was correlated with Fluoro-Jade B-positive degenerating neurons (Fig. 4M-4O). Control brains showed no evidence of DNA fragmentation or neuronal degeneration (Fig. 4D and 4L). Neurons (60%) comprised the majority of cell type with DNA fragmentation followed by astrocytes (26%), and other cells including oligodendrocytes (14%) (Fig. 4H-4K and 4P-4S). Fragmented DNA was rarely found in endothelial cell layer of blood vessels or microglia, which appeared relatively spared from injury (red arrows, Fig. 4I). Thickened microglial processes wrapped around the cell bodies as if engulfing fragmented DNA of disintegrating neurons and astrocytes (Fig. 4R4). The amount of DNA fragmentation both in the cortex and white matter was significantly correlated with the seizure frequency prior to surgery (*p *< 0.003) (Fig. 4C). The other clinical variables, such as age of onset of seizure, duration of seizures, presence of febrile seizure, family history of epilepsy, mental retardation or hemiparesis, however, did not show a significant correlation with DNA fragmentation.

### Increased levels of proinflammatory cytokines and chemokines in the resected cortices from pediatric epilepsy patients

**Figure 5:**
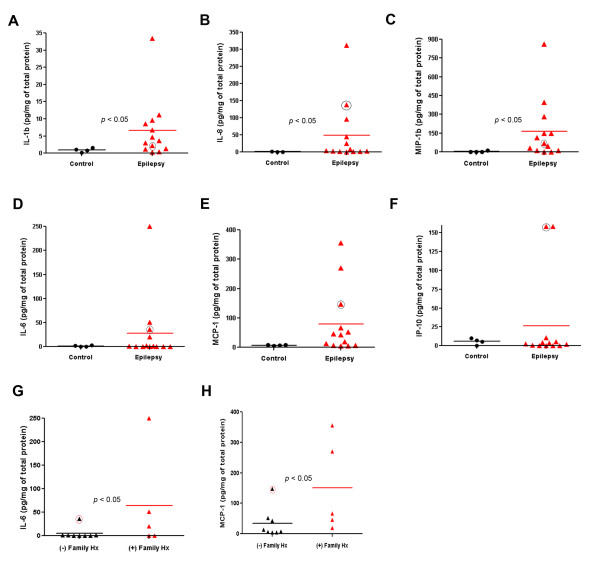
**Cytokine analysis by ELISA**. IL-1β (**A**), IL-8 (**B**) and MIP-1β (**C**) are significantly increased in brains of patients with epilepsy (*p *< 0.05) whereas. IL-6 (**D**), MCP-1 (**E**) and IP-10 (**F**) show sporadic and variable increases in our patients with epilepsy. Note that some of our patients show highly elevated cytokine release comparable to Rasmussen's encephalitis (black circle). IL-6 (**G**) and MCP-1 (**H**) are significantly increased in brains from patients with a family history of epilepsy (*p *< 0.05). Rasmussen's encephalitis patient (black circle) is the only one who shows high IL-6 and MCP-1 levels among the patients without family history of epilepsy.

IL-1β, IL-8, IL-12p70 and MIP-1β were significantly increased in the cortex of our patients (Fig. 5) (*p *< 0.05). IL-6, MCP-1, IP-10, and MDC were increased in some, but not all patients. A few patients showed markedly elevated IL-1β or IL-6 levels, even higher than levels in the patient with Rasmussen's encephalitis (black circle in Fig. 5). There was a significant correlation between a family history of epilepsy and specific cytokine levels: IL-6 and MCP-1 were higher in patients with family history of epilepsy than sporadic cases (Fig. 5G and 5H) (*p *< 0.05). Notably, IL-6 and MCP-1 were elevated significantly in the patient with Rasmussen's encephalitis (black circle in Fig. 5G and 5H) uniquely among the sporadic cases.

## Discussion

The main findings of present study are the demonstration of abundant cell injury, marked glial activation and neuroinflammation in the surgically resected cortical tissue from patients with intractable epilepsy. Dense, extensive, and wide-spread fragmented DNA was found both in the cortex and in white matter of the resected brain tissue. Panlaminar astrocytosis, diffuse microglial activation and release of proinflammatory cytokines and chemokines consistent with chronic and sustained neuroinflammatory responses were present in the epileptogenic lesions. It appears that intractable epilepsy in childhood, regardless of the cause, age, history of febrile seizure or family history of epilepsy, is marked by ongoing active cell injury and glial activation. What used to be considered a hallmark of Rasmussen's encephalitis - neuronal injury and microglial activation - is also noted in our cases of focal cortical dysplasia and encephalomalacia, two common causes of intractable childhood epilepsy.

Microglial activation and proliferation have been demonstrated in sclerotic hippocampal tissue [26], within the dysplastric cortex of focal cortical dysplasia [5], and within and around the tumor bed in highly epileptogenic gangliogliomas and dysembryoplastic neuroepithelial tumors [6]. The density of activated microglia correlated both with the duration of epilepsy and with the seizure frequency [5,6].

Although our study deals almost exclusively with children - and adult epilepsy may differ fundamentally in its pathophysiology including the contribution of inflammation - our results, together with those from other studies, suggest that activation of microglia is an important associated feature of chronic intractable epilepsy.

In our study, astrocytic activation was particularly striking in the cortex; the infiltrative astrogliosis was associated with up-regulation of selective cytokines. Although astroglial proliferation is a well-known accompaniment of a sclerotic hippocampus in adult patients with temporal lobe epilepsy [27], a causative role of activated astrocytes in ictogenesis, seizure maintenance and epileptogenesis has only recently been recognized. Direct stimulation of astrocytes is sufficient to cause a paroxysmal depolarization shift (PDS) and neuronal synchronization in acute seizure models [28]. Activated astrocytes display distinct changes in glial membrane channels and receptors to promote neuronal hyperexcitability and seizure generation [29].

A massive inflammatory response induced by seizures and interactions between microglia, astrocytes and neurons may be an important component in epileptogenesis. Complex cross-talks between microglia and astrocyte during neuroinflammatory insults can influence glutamate-dependent responses and immune regulatory role of astrocytes [30]. All our patients showed diffuse astrocytosis in the epileptogenic cortices, and these proliferative astrocytes were found in close proximity to activated microglia and neurons, strategically positioned to allow such cross-talk.

Reactive microglia and astrocytes provide a rich source of cytokines after injury or insult [31]. Cytokines, in turn, can influence the activation of astrocytes and microglia. Injection of pro-inflammatory cytokines, for example, can induce astrogliosis in healthy animals [32], while injury-induced microglial activation is suppressed in TNF receptor-knockout mice [33]. Causative role of cytokines in epileptogenesis remains to be elucidated. Cytokines may contribute initially perhaps by inciting seizures in the developing brain after being induced by seizures or tissue injury, exacerbate tissue injury and promote further seizures. Alternatively, neuroinflammation may be a mere byproduct of epilepsy, rather than causally related. Pre-treatment with IL-1 receptor antagonists was recently shown to significantly reduce blood brain barrier (BBB) disruption and delay the onset of status epilepticus in the lithium-pilocarpine seizure models [34], further supporting the contribution of cytokines and BBB damage to epilepsy. Furthermore, cytokine gene polymorphisms have been linked to epilepsy susceptibility. The increased frequency of biallelic polymorphisms in the promoter region of IL-1β at the -511 position were reported in patients with temporal lobe epilepsy with hippocampal sclerosis, and in prolonged febrile convulsion [35]. We found significantly higher IL-6 and MCP-1 level in our small number of patients with a family history of epilepsy. It may be worthwhile to explore further a possible link between chronic epilepsy and genetic susceptibility to inflammation.

Investigations in the most extreme case of seizure activity - status epilepticus - in both human and animals demonstrate that prolonged seizures can cause neuronal death [36,37]. Glutamate-mediated excitotoxicity, necrosis and activation of apoptosis are contributing mechanisms [38,39]. Thus the finding that frequent discrete seizures in our patients may have caused both immediate necrotic and delayed apoptotic cell death was not surprising. An unexpected finding was the degree and extent of the cell injury seen in our pediatric cases in contrast to adult patients with TLE [25]. Necrosis, often TUNEL-positive, is the main form of seizure-induced cell death and is known to induce an inflammatory response. Therefore, glial reaction may be a result of cell death as well as a trigger. Interestingly, DNA fragmentation was not correlated with the magnitude of glial reaction in our study raising the possibility that frequent seizures may be responsible for both cell death and glial reaction. Seizure-induced cognitive impairments are influenced by the extent of damage caused by seizures in animal models [40]. This result is supported by our finding that majority of our patients with abundant cell injury have mental retardation. It appears that intractable epilepsy in childhood due to focal cortical dysplasia and encephalomalacia as well as Rasmussen's encephalitis are marked by ongoing active cell injury.

While the majority of the cell population showing DNA fragmentation in the cortex are neurons, we found that over 25% of DNA fragmentation was due to astroglial damage. In children having recent seizures, CSF GFAP levels were significantly elevated within 24 hours, and correlated with seizure duration, suggesting that astrocytic injury may occur primarily during prolonged seizures [41]. Astrocytes are thought to be more resistant to injury compared to neurons and oligodendrocytes, which are both vulnerable to glutamate-mediated death [42]. At 6-24 hours after status epilepticus, however, TUNEL-positive astrocytes and neurons are increased and then subsequently reduced after 7-14 days due to phagocytosis by activated microglia/macrophages [43].

In summary, neuroinflammation and ongoing cell injury were extensive and wide-spread in our patients with intractable epilepsy. Although it is not possible to infer causality from descriptive human studies, our data suggest that increased seizure frequency is associated with increased neuronal and astrocytic injury, and that frequent seizures are also associated with massive glial activation and inflammatory responses in the epileptogenic cortex. Microglial activation, astocytic proliferation, and proinflammatory cytokine production may promote seizures, further exacerbate epilepsy and cause subsequent degeneration of neurons, astrocytes and oligodendrocytes. If so, there may be a potential role for anti-inflammatory therapy targeting activated astrocytes and microglia as a novel therapeutic strategy to prevent or limit epileptogenesis and cell injury associated with seizures in the vulnerable developing nervous system.

## Competing interests

The authors declare that they have no competing interests.

## Authors' contributions

JC processed brain tissues from the patients in the operating room, performed immunohistochemistry and immunofluorescence staining and confocal micoroscopy, conducted multiplex analysis of cytokines and chemokines from the protein extracts of patients' brain tissues, analysis the data and drafted the manuscript. DN, LL, KK and SS decided epilepsy surgery and collected the demographic and postoperative outcome data from the patients and helped in drafting and preparing the manuscript for publication. TA, AD and JR did operation and removed epileptogenic lesions from the patients. VR examined and reviewed epileptogenic brain tissue slides and also helped in drafting and preparing the manuscript for publication. SK reviewed and helped in analyzing data, obtained IRB approval and permission from the patients and their parents and also helped in drafting and preparing the manuscript for publication.
